# A Comparison of Central Venous Access to the Internal Jugular Vein and Two Standard Approaches to the Subclavian Vein: A Study of Cross-Sectional Areas Using Computed Tomography Scans

**DOI:** 10.7759/cureus.23823

**Published:** 2022-04-04

**Authors:** Joshua W Sappenfield, Lou Ann Cooper, Robert Evans Heithaus, Samsun Lampotang

**Affiliations:** 1 Anesthesiology, University of Florida, Gainesville, USA; 2 College of Medicine, University of Florida, Gainesville, USA; 3 Radiology, Medical Center Radiology Group, Orlando, USA

**Keywords:** radiologists, thyroid cartilage, ultrasonography, subclavian vein, jugular vein

## Abstract

Introduction

The supraclavicular approach to the subclavian vein has been cited as having many advantages to the infraclavicular approach, including a larger short-axis cross-sectional area, a greater margin of safety, and fewer complications.

Methods

To examine whether a larger short-axis cross-sectional area of the subclavian vein at the supraclavicular fossa is a potential explanation for the reduction in attempts with the supraclavicular approach seen in a previous study, we examined computed tomography scans from 50 patients (24 M, 26 F). The short-axis cross-sectional areas of the subclavian vein at the mid-clavicular line, the subclavian vein in the supraclavicular fossa, and the internal jugular vein at the level of the thyroid cartilage were calculated.

Results

The internal jugular vein short-axis cross-sectional area was significantly larger than the subclavian vein short-axis cross-sections measured at each location. We found no difference between the short-axis cross-sectional areas of the subclavian vein or when comparing measurements as a factor of gender, age, or race. Weight had a significant relationship to the short-axis cross-sectional area of the internal jugular vein and subclavian vein at the mid-clavicular vein.

Conclusions

On supine computed tomographic imaging, the subclavian vein short-axis cross-section was not larger in the supraclavicular fossa than the mid-clavicular line. The short-axis cross-sectional area of the subclavian vein at the supraclavicular fossa does not appear to contribute to the decrease in attempts to access it. Weight, but not necessarily height, appears to be correlated with central vein size.

## Introduction

In 1965, Yoffa [1] described the supraclavicular approach to the subclavian vein. This approach, compared to the infraclavicular approach, has been shown to provide a larger, more superficial short-axis target cross-sectional area (CSA), a greater margin of safety from lung pleura, less malpositioning, and possibly fewer complications [2-4]. Some complications are more serious and may require additional surgery for correction [5]. Sappenfield et al. [6] observed a statistically significant reduction in attempts to obtain central venous access using the supraclavicular approach to the subclavian vein compared to the infraclavicular approach in a simulator-based study. A reduction in time to access the subclavian vein using the supraclavicular approach (without ultrasound (US)) compared not only to the infraclavicular approach but also to US-guided access to the internal jugular (IJ) vein was also noted [6]. Furthermore, Sappenfield et al. described a US approach using a 3-cm, 13- to 6-MHz US probe to reliably access the subclavian vein. In this single volunteer study, the short-axis CSA was more than double the short-axis CSAs of the other two vein locations. Based on these observations, we hypothesized that the short-axis CSA of the subclavian vein at the supraclavicular fossa (SF) was larger than at the mid-clavicular line and the IJ at the level of the thyroid cartilage.

## Materials and methods

This study was given University of Florida Institutional Review Board (IRB) approval (IRB201800669), and written consent was waived by the IRB. With few published data on comparative vein size, we used a power analysis/sample size calculation for the average within-subject difference in short-axis cross-sectional venous measurements. We assumed a paired t-test, medium effect size (0.50 standard deviation units), a relatively small correlation between measurements (0.25), a two-tailed test, significance level of α = 0.05, and target power = 0.80 (http://plaza.ufl.edu/algina/pwr.within.factors.sas). Based on these considerations, our target sample size was n = 50.

Inclusion criteria included having a computed tomography (CT) angiogram of the neck, CT venogram of the neck, CT angiogram of the right upper extremity, or a CT venogram of the chest between March 20, 2017, and March 30, 2018. Exclusion criteria included CT angiograms where contrast bolus obscured the intimal boundaries of the vein, current incarcerated inmate, and incomplete data in the medical record. A convenience sample of 59 patients was enrolled with 50 patient records submitted for data analysis. Six patients were excluded because of the inability to measure the subclavian vein, one patient was excluded because he was a prison inmate, and two patients were excluded for incomplete data.

**Figure 1:**
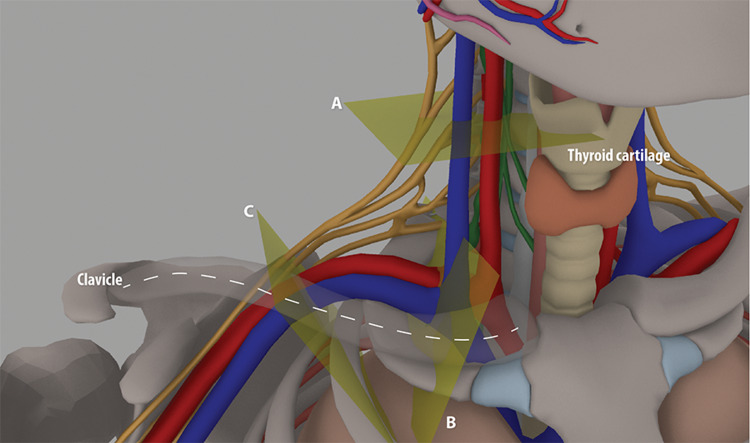
Cross-sectional planes through veins where measurements were taken (A) The internal jugular vein at the thyroid cartilage; (B) the subclavian vein at the supraclavicular fossa; (C) the subclavian vein at the midclavicle.

The radiographic studies were viewed in picture archiving and communication systems (PACS) (Visage Imaging Inc., San Diego, CA). In each of the studies, patients had their right arm down by their side. The short-axis diameters were measured of the subclavian vein at the SF (Figure 1, cross-section B), the subclavian vein at the midclavicle (Figure 1, cross-section C), and the IJ at the thyroid cartilage (Figure 1, cross-section A). Measurements were obtained in two orthogonal planes using the ruler/measurement function in PACS. One-half the length of the two axes was used as the short (α) and long (β) radii for calculating the CSA of the veins, assuming ellipsoid shape; A = παβ. When a vascular valve was encountered in between the clavicle and the first rib, the measurement of the subclavian vein was obtained from a cross-section either proximal or distal to the valve at the point that was visually just outside the dilated portion of the vein. If the external jugular vein ostium was associated with the junction of the IJ and the subclavian vein, resulting in a vessel trifurcation, the subclavian vein diameter measurement was obtained distal to the origin of the external jugular and not at the junction of the IJ and subclavian vein at the brachiocephalic vein.

We recorded the distance, at the deepest skin crease, from the skin to the subclavian vein in the SF. Age, race, gender, height, weight, and body mass index (BMI) were recorded. For patients where one of height, weight, or BMI was missing, it was calculated from the other two variables from this formula: BMI = weight/height^2^.

Data analysis was conducted using SAS, version 9.4 (SAS Institute Inc., Cary, NC). The Pearson correlations between venous CSA measurements and study variables were computed. The 95% confidence interval for the correlation estimates was calculated using Fisher’s z-transformation. Because the three cross-sectional venous areas were measured within the same person, paired t-tests on the difference in CSA between the SF (CSA SF) versus mid-clavicular line (CSA MCL), CSA SF versus CSA IJ, and CSA MCL versus CSA IJ were calculated. In addition, independent sample t-tests were computed for outcome variables based on gender and race.

## Results

Bivariate correlations between venous CSA measurements and study variables are reported in Table 1. The correlation coefficient, r, is an effect size measure. Cohen [7] provides rules of thumb for interpreting these effect sizes, suggesting that an r of |0.1| represents a “small” effect size, |0.3| represents a “medium” effect size, and |0.5| represents a “large” effect size. The confidence interval is a range that contains the true correlation with 95% confidence. For CSA SF, the only significant correlation was between CSA SF and CSA MCL, r = 0.38 (0.11, 0.59). This means that the size of the subclavian vein at the MCL was related to the size of the subclavian vein at the SF. In addition to an association with CSA SF, CSA MCL was significantly correlated with weight, r = 0.38 (0.12, 0.60) and BMI, r = 0.36 (0.09, 0.58). Thus, patient habitus was associated with the size of the subclavian vein. In contrast, CSA IJ was also significantly correlated with weight, r = 0.40 (0.13, 0.61), but, interestingly, not to BMI, r = 0.26 (-0.02, 0.50). The distance from the SF to the subclavian vein was significantly correlated with weight, r = 0.42 (0.16, 0.63) and BMI, r = 0.37 (0.10, 0.59). Because the correlation between height and BMI was not significant, the correlation between venous short-axis CSA measurements and BMI would seem to be largely due to the contribution of weight to the calculation of BMI.

**Table 1:** Relationships between venous cross-sectional area measurements on CT scan CSA: cross-sectional area in cm^2^; CT: computed tomography; IJ: internal jugular; MCL: mid-clavicular line; SCV: subclavian vein; SF: supraclavicular fossa. r is the estimated Pearson Product Moment correlation. The 95% CI was calculated using Fisher's Z-transformation. *Significant at α = 0.05. This tests the null hypothesis that the correlation in the population is 0.

Variable	With variable	r	95% confidence interval	p-value for H0; ρ=0*
CSA SF	CSA MCL	0.38	(0.11, 0.59)	0.006*
CSA SF	CSA IJ	0.10	(–0.18, 0.37)	0.477
CSA SF	Distance SF to SCV (cm)	–0.10	(­–0.37, 0.18)	0.486
CSA MCL	CSA IJ	0.17	(­–0.12, 0.43)	0.239
CSA MCL	Distance SF to SCV (cm)	0.24	(­–0.04, 0.49)	0.087
CSA IJ	Distance SF to SCV (cm)	0.27	(­–0.01, 0.51)	0.055

Study variables and descriptive statistics for the study participants are reported in Table 2. The difference between short-axis CSA measures was computed for each participant and the dependent samples t-test was then computed. The short-axis CSA of the IJ was significantly greater than CSA measures at both the MCL (t = 6.70, p < 0.0001) and at the SF (t = 6.70, p < 0.0001); there was no difference between short-axis CSA SF and short-axis CSA MCL (t = −0.64, p = 0.53). The short-axis CSA IJ was nearly twice as large as measurements at the SF and MCL.

**Table 2:** Outcome measures: cross-sectional area of veins based on CT scans, age, height, weight, and BMI *Dependent samples (paired) t-test (within subjects). CT: computed tomography; BMI: body mass index; CSA: cross-sectional area; IJ: internal jugular; SF: supra fossa; MCL: mid-clavicular line.

	N	Mean	SD	95% confidence interval	t^1^	p
Cross-sectional area (CSA) in cm^2^						
Supra fossa (SF)	50	1.24	0.33	(1.15, 1.34)		
Mid-clavicular line (MCL)	50	1.28	0.34	(1.18, 1.37)		
Internal jugular (IJ)	50	2.22	1.00	(1.94, 2.50)		
Distance SF to subclavian vein (cm)	50	2.68	0.84	(2.44, 2.92)		
Age (years)	50	61.90	17.25	(57.00, 66.80)		
Height (m)	50	1.70	0.10	(1.67, 1.72)		
Weight (kg)	50	80.02	18.50	(74.76, 85.28)		
BMI (kg/m^2^)	50	27.27	6.87	(25.31, 29.22)		
CSA ratio						
IJ/SF	50	1.87	0.93	(1.61, 2.14)		
IJ/MCL	50	1.81	0.87	(1.56, 2.06)		
SF/MCL	50	1.02	0.36	(0.92, 1.12)		
CSA differences (cm^2^)						
IJ - SF	50	0.98	1.02	(0.69, 1.27)	6.80	<0.0001
IJ - MCL	50	0.94	0.99	(0.66, 1.22)	6.70	<0.0001
SF - MCL	50	–0.04	0.37	(–0.14, 0.07)	–0.64	0.53

Independent sample t-tests using the Satterthwaite method were conducted to examine the possibility of gender and race differences. Satterthwaite is an alternative to the pooled-variance t-test and is used when the assumption that the two populations have equal variances seems unreasonable. As shown in Table 3, there were no statistically significant differences found between the CSA of the central veins based on race or gender. No gender differences were seen in weight or BMI; however, on average, males were found to be taller than females (t = 8.03, p < 0.0001). After eliminating one participant with no race identified, there were no differences between the African-American (n = 9) and Caucasian (n = 40) patients on any of the variables examined in this study.

**Table 3:** Venous cross-sectional area measurements from CT scans by gender and race ^a^One participant identified as Hispanic (ethnicity) with no race and was omitted from this analysis. SCV: subclavian vein; SF: supra fossa; IJ: internal jugular; SF: supra fossa; MCL: mid-clavicular line; BMI: body mass index

	Gender					
	Female (n = 26)		Male (n = 24)			
	Mean	SD	Mean	SD	t	p-value
Supra fossa (SF) in cm^2^	1.24	0.36	1.25	0.30	–0.12	0.90
Mid-clavicular line (MCL) in cm^2^	1.23	0.34	1.33	0.34	–0.99	0.33
Internal jugular (IJ) in cm^2^	2.24	0.93	2.20	1.08	0.13	0.89
Distance SF to SCV (cm)	2.62	0.87	2.73	0.83	–0.46	0.65
Age (years)	64.23	17.82	59.38	16.61	1.00	0.32
BMI (kg/m^2^)	28.59	7.00	25.84	6.58	1.43	0.16
Height (m)	1.62	0.05	1.77	0.08	–8.00	<0.0001
Weight (kg)	75.45	19.77	84.98	15.98	–1.88	0.07
	Race^a^					
	African-American (n = 9)		Caucasian (n = 40)			
	Mean	SD	Mean	SD	t	p
Supra fossa (SF) in cm^2^	1.32	0.40	1.22	0.32	0.70	0.50
Mid-clavicular line (MCL) in cm^2^	1.40	0.24	1.24	0.35	1.66	0.12
Internal jugular (IJ) in cm^2^	2.44	0.90	2.18	1.03	0.78	0.45
Distance SF to SCV (cm)	2.93	0.85	2.63	0.85	0.97	0.35
Age (years)	56.00	9.49	63.80	18.17	–1.83	0.08
BMI (kg/m^2^)	32.19	8.40	26.39	6.02	1.96	0.08
Height (m)	1.69	0.12	1.69	0.09	–0.49	0.85
Weight (kg)	89.84	18.94	78.28	17.96	1.67	0.12

## Discussion

Based on the findings in this study, we failed to prove our hypothesis that the subclavian vein short-axis CSA in the SF is larger than at the MCL or the IJ vein. This hypothesis was based on the findings by Sappenfield et al. [6] of the lower complication rate in a simulator-based study and viewing the larger CSA with US on a single volunteer.

Interestingly, in this study, about one-third of the patients had a valve at the MCL inside their subclavian vein. This valve made the short-axis CSA of the vessel substantially larger compared to more medially or laterally. This would potentially make it easier for an operator to access the vessel at the location of the valve. On the flip side, the valves in this area can increase the difficulty of placing a line using the Seldinger technique because it may be hard to move the wire through the valve. In this study, no valves were seen on the subclavian vein near the junction with the innominate vein.

There are limitations to this study. First, none of these patients were in Trendelenburg but were horizontal for their CT scan. The Trendelenburg position may cause the larger veins to swell with the increase in hydrostatic pressure. However, this effect may be negligible [8,9]. Second, none of these patients were actually having a central line placed, so all measurements were made based on hypothetical approaches to accessing the central veins. It is highly likely that an operator, e.g., an anesthesiologist, who is performing the procedure using US may have a different insonation angle to the vein than what was measured by the radiologist who can easily see the entire course of the subclavian vein in one image. The proceduralist may even choose an oblique cross-section of the vein as the target. Both of these pragmatic differences would potentially change the CSA of the vein at the point of access. For example, the plane through the IJ vein at the level of the thyroid cartilage was preferred as the study location because the access point is most likely to be chosen by the average operator when obtaining central venous access using a landmark technique. However, Giordano et al. [10] demonstrated that the IJ vein is wider the more proximal it is. Thus, the IJ site, which was found to be larger than the other two sites in this study, would potentially be even larger at the point it would normally be accessed by a proceduralist. Following this logic, a potential better target for accessing the central veins would be the confluence of the IJ and subclavian veins, the innominate vein. This target would necessarily have an even larger CSA.

## Conclusions

This study failed to confirm that the subclavian vein has a larger short-axis CSA in the SF compared to the subclavian vein at the MCL, as well as compared to the IJ vein. Thus, this study does not support that a larger short-axis CSA at the SF is the reason that a lower complication rate was observed in simulated venous access. Further studies are necessary to confirm the lower complication rate, as well as to investigate other potential techniques to find the optimal US window for an approach to obtain central venous access.

## References

[1] Yoffa D (1965). Supraclavicular subclavian venipuncture and catheterisation. Lancet.

[2] Patrick SP, Tijunelis MA, Johnson S, Herbert ME (2009). Supraclavicular subclavian vein catheterization: the forgotten central line. West J Emerg Med.

[3] Conroy JM, Rajagopalan PR, Baker JD 3rd, Bailey MK (1990). A modification of the supraclavicular approach to the central circulation. South Med J.

[4] Dronen S, Thompson B, Nowak R, Tomlanovich M (1982). Subclavian vein catheterization during cardiopulmonary resuscitation: a prospective comparison of the supraclavicular and infraclavicular percutaneous approaches. JAMA.

[5] Sasson M, Montorfano L, Bordes SJ, Sarmiento Cobos M, Grove M (2021). Subclavian artery injury following central venous catheter placement. Cureus.

[6] Sappenfield J, Grek S, Cooper LA, Lizdas DE, Lampotang S (2019). Reduced complications of supraclavicular approach in simulated central venous access: applicability to military medicine. Mil Med.

[7] Cohen J (1988). Statistical power analysis for the behavioral sciences, 2nd ed. https://www.taylorfrancis.com/books/mono/10.4324/9780203771587/statistical-power-analysis-behavioral-sciences-jacob-cohen.

[8] Nassar B, Deol GR, Ashby A, Collett N, Schmidt GA (2013). Trendelenburg position does not increase cross-sectional area of the internal jugular vein predictably. Chest.

[9] Botero M, White SE, Younginer JG, Lobato EB (2001). Effects of Trendelenburg position and positive intrathoracic pressure on internal jugular vein cross-sectional area in anesthetized children. J Clin Anesth.

[10] Giordano CR, Murtagh KR, Mills J, Deitte LA, Rice MJ, Tighe PJ (2016). Locating the optimal internal jugular target site for central venous line placement. J Clin Anesth.

